# Impact of a 16‐week strength training program on physical performance, body composition and cardiac remodeling in previously untrained women and men

**DOI:** 10.1002/ejsc.12033

**Published:** 2024-03-18

**Authors:** Antoine Grandperrin, Pierre Ollive, Yanis Kretel, Claire Maufrais, Stéphane Nottin

**Affiliations:** ^1^ Avignon University LaPEC EA 4278 Avignon France; ^2^ YAKHA Sport Entraigues‐sur‐la‐Sorgue France

**Keywords:** body composition, cardiovascular health, strength‐training, women

## Abstract

Even if more and more women are involved in strength‐training (ST) programs in fitness centers, studies on strength gain, body composition, and cardiac remodeling were mainly conducted in men and whether they are similar in women remains to be explored. In this context, the aim of our study was to assess the effect of a supervised ST program on strength gains, body composition, and cardiac remodeling in previously untrained women and men. 17 healthy and previously untrained young women and 17 young men participated in a supervised 16‐week ST program built according to the recommendation of the American College of Sports Medicine in terms of intensity, and strictly using similar volume and intensity in both groups. Strength performance, body composition, and cardiac remodeling were evaluated every 4 weeks. Cardiac adaptations were assessed using resting echocardiography, including regional 2D‐Strain analysis of the left atrium and ventricle (LA and LV, respectively). Despite lower values at baseline, women exhibited similar or even higher strength gains compared to men. ST induced a decrease of body and abdominal fat mass and an increase of lean body mass in both groups. Similar cardiac remodeling was observed in women, and women, including an early and progressive LV and LA enlargement throughout the ST program, without any alteration of LV diastolic and systolic functions. These findings underlie that ST programs are highly suitable for women to enhance their strength performance and their cardiovascular health.

## Highlights

- After 16 weeks of a supervised strength‐training program, designed according to recommendations of the American College of Sports Medicine, previously untrained women increased their strength performance to a similar extent compared to men.
- 16 weeks of strength‐training, performed at high intensity and mobilizing important muscle mass, induces positive alteration of the body composition, characterized by an increase of lean body mass and a decrease of total and abdominal fat mass, suggesting cardiovascular health benefits, in both women and men.
- Strength‐training induced an early and progressive left ventricular and left atrial enlargement in both women and men, without any cardiac functional alterations. In all, our results highlighted that strength‐training is highly suitable to enhance the strength performance and the cardiovascular health of women, in a similar manner to those previously observed in men.

## 1 INTRODUCTION

Given that there are over 60,000 fitness centers across Europe with a total membership of more than 60 million people, strength training (ST) represents a popular physical activity (Taaffe et al., 2006). Although women were historically banished from this male‐dominated environment, the expansion of fitness centers has substantially changed mentalities. A recent epidemiological study carried out throughout 28 countries reported that women were equally involved in ST as men (Bennie et al., 2020). Women involved in fitness centers performed ST not only for esthetic appearance but also to enhance strength performance, attenuate the age‐related declines in muscle strength and muscle mass, and to maintain cardiovascular health (Taaffe et al., 2006).

The review of Westcott (2012) provides evidence that ST is effective in enhancing several important aspects of physical health. Indeed, in addition to strength and power adaptations largely reported in the scientific literature (see for review (Deschenes & Kraemer, 2002)), ST also alters body composition, with an increase of lean body mass and a decrease of body fat mass (see for review (Kraemer et al., 2002)). Moreover, when the training load is sufficient, ST leads progressively to an exercise‐induced cardiac remodeling (ECIR) (Pelliccia et al., 2012). ECIR in ST athletes results mainly from short but intense bouts of increased peripheral vascular resistance causing transient but marked increases of systolic left ventricular pressure (MacDougall et al., 1992; Pelliccia et al., 2012).

Despite the growing interest of women for ST, their capability to respond to ST in a similar extent compared to men in regards to many factors such as strength performance, body composition, or cardiac remodeling remains not well‐established. Even if long term ST results in less muscle gains in women compared to men, mainly attributed to lower testosterone levels in female (Weiss et al., 1983), the sex specificities in response to longitudinal ST in previously untrained subjects remain poorly investigated. The 10‐fold lower concentrations of testosterone reported in women (Weiss et al., 1983) probably lead to lower stimulation of hypertrophy signaling pathway and consequently lower muscle and strength gains consecutive to training. However, these considerations need to be confirmed using a well‐designed protocol. Indeed, most of the studies were conducted in men only, and to our knowledge, only few studies focused on women. Moreover, these studies reported conflicting results (See (Roberts et al., 2020) for review) probably due to confounding factors such as heterogeneous levels of practice before inclusion or different training programs in terms of exercise intensity and/or duration (George et al., 1998; Haykowsky et al., 1998; Spence et al., 2011; Wernstedt et al., 2002). To overcome these limitations, longitudinal studies, including supervised ST programs based on exercises of similar relative intensities in women and men, are needed, since it is now well‐known that the parameters of the ST are essentials to promote adaptations. The American college of Sports medicine (ACSM) (Garber et al., 2011) published recommendations (i.e., training intensity, training volume, training frequency, and type of exercise) to improve the efficiency of ST programs, but only few studies matched with these recommendations (Häkkinen, Kallinen et al., 1998, Häkkinen, Pakarinen et al., 1998; Lemmer et al., 2000). Finally, cardiac adaptations following ST in previously untrained women and men was poorly investigated and the few studies conducted in the field mainly focused on LV morphological parameters. Data are scarce regarding left ventricular (LV) and left atrial (LA) functional remodeling. On the last decade, advances in echocardiography based on 2D‐strain have provided the possibility to assess regional LV strain, a more sensitive parameters to evaluate cardiac function in various conditions. More recently, non‐invasive parameters for the assessment of LA function using echocardiography were developed (Vieira et al., 2014). Briefly, the LA is a complex and active chamber, which acts as a reservoir during ventricular systole and favors ventricular filling during diastole by its role as a conduit and booster pump (Grandperrin, Schuster, et al., 2022). To the best of our knowledge, the evaluation of LV and LA function using 2D‐strain echocardiography during longitudinal ST was never performed in previously untrained subjects.

In this context, the aim of the present study was to assess the effects of a supervised ST program on strength gains, body composition, and cardiac remodeling in previously untrained women and men. We used resting echocardiography based on 2D‐strain analysis to evaluate left ventricular (LV) and left atrial (LA) myocardial regional function. We hypothesized that, when ST is built in accordance with the ACSM recommendations and with similar relative training loads, women would respond to a similar extend in terms of strength improvements, body composition, and cardiac remodeling compared to men.

## 2 METHODS

This study used a 16‐week longitudinal supervised ST program. Assessments of strength performance, body composition and cardiac morphology, and function were conducted before the beginning of the ST program and then every 4 weeks in order to consider the increase of the muscular strength and therefore to adjust training intensities during the ST program.

### 2.1 Study population

**FIGURE 1:**
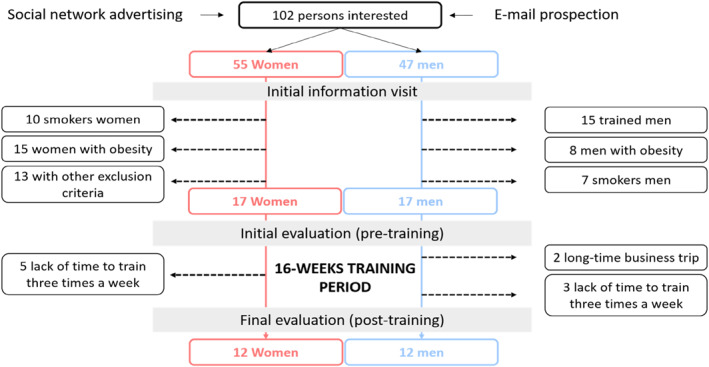
Flow chart of patient recruitment with inclusion and exclusion criteria consideration.

In partnership with French gymnasiums, subjects were recruited from announcement on social networks. Subjects were then rigorously included in accordance with our inclusion criteria. Thirty‐four participants aged 18–40 years were recruited (17 men and 17 women). None of the participants practiced more than 1 hour of physical training (aerobic or strength training) per week in the 3 years preceding entry into the study's protocol. The physical activity level of each participant was assessed using the 16‐item Global Physical Activity Questionnaire version 2 adapted (GPAQ‐2), which collects information on sedentary behavior and physical activity in three domains: activity at work, travel to and from places, and recreational activities during a typical week (Armstrong et al., 2006). Exclusion criteria were a history of coronary artery disease, congestive heart failure, moderate or severe valvular heart disease, congenital heart disease, heart failure, LV systolic dysfunction (defined as an ejection fraction <50% on echocardiography), hypertension (blood pressure of >140/90 mmHg or the use of antihypertensive medication), respiratory disease, metabolic disorders, and/or cigarette smoking in the past 10 years. None of participants were obese (i.e., body mass index >30 kg.m^−2^) and were taking any recreational drugs or medications. The study was approved by the French ethics committee for sports science (IRB 00012476‐2020‐10‐10‐67). Before taking part in the study, the subjects were informed of the benefits and risks of the investigation prior to signing an institutionally approved informed consent document to participate in the study. The Figure 1 represents the flow chart of our study population with the recruitment process.

### 2.2 Training protocol

The training protocol was designed according to the ACSM's recommendations for ST in healthy adults (Garber et al., 2011) and supervised by three experienced instructors (P.O., Y.K., and A.G.). It included three training sessions (predominantly performed on Monday, Thursday, and Saturday to respect recovery time) per week over a 16‐week period. Each session began with a cardiorespiratory warm up performed on a treadmill or on a bike followed by an articular and muscular warm‐up. All strength exercises were performed at 70% of the participant's individual one‐repetition maximum (1‐RM; i.e., the maximum amount of weight that the participant can pull or push in one repetition). In line with the ACSM guidelines, we used principally poly‐articular exercises. Each session targeted, respectively, the inferior muscle groups (squat, leg‐press, leg extension, and leg‐curl), the trunk (bench press, incline bench press, butterfly, vertical pull‐ups, and horizontal rowing), and the arms and shoulders (military press, side elevations, front elevations, bicep curls with dumbbells, bicep curls with EZ bar, and triceps curls with rope extension). For each exercise, the participant's performed four sets of 10 repetitions with a resting period of 90 s between each set. Each training session lasted approximatively 90 min. Participants were asked to not perform any other physical training sessions. The participants were included in the final analyses if they performed more than 90% of the training sessions. Of note, all women included in the study used oral contraceptive without interruption during the training program.

### 2.3 Anthropometric and performances parameters

Body mass (kg) and body composition (i.e., lean body mass, kg; body fat mass, %; and abdominal fat mass, kg) were assessed using an eight‐electrodes multifrequency bioelectrical impedance device (Inbody 270, France) validated against dual energy x‐ray absorptiometry. Measures were performed at the same time of day, in underwear, without water or food consumption, respectively, 1 and 3 h before, in order to respect recommendations of the manufacturer and to standardize acquisitions. Body surface area was assessed according to Dubois and Dubois formula (Villa et al., 2017). The 1‐RM was assessed on bench press and back squat using incremental loads method as previously described (Masamoto et al., 2003). Briefly, after a general warm‐up of low‐intensity aerobic exercise (5 min on a bike and 5 min on a rowing machine), subjects performed series of submaximal sets of 8, 5, and 2 repetitions with increasing loads until their 1‐RM. The subjects rested for at least 4 min between the trials and the 1‐RM was recorded as the maximum resistance that could be lifted through the full range of motion.

### 2.4 Echocardiographic recordings

Participants were examined in the fasting state after a resting period of at least 30 min. Prior to the cardiac evaluation, blood pressure was evaluated using an automated sphygmomanometer. Echocardiography was performed on the participant in the left lateral decubitus position using the Vivid Q system (GE Healthcare, Horten, Norway) and a 3.5‐MHz transducer (M4S probe). Cine loops were recorded in the parasternal short axis (basal and apical levels), the parasternal long axis and the apical five, four, two and three chambers views and saved for a blinded offline analysis (EchoPAC, BT113 version, GE Healthcare). Grayscale images were saved at a frame rate of 80–90 frames per second and color tissue velocity images at a frame rate of 120–140 frames per second. All measurements were averaged over three to five cardiac cycles. Two‐dimension echocardiographic measurements were performed in accordance with the guidelines from the American Society of Echocardiography (Lang et al., 2015). Resting echocardiography measurements were performed at the same time of day for each participant.

### 2.5 Echocardiographic analysis

#### 2.5.1 Left ventricular morphology and global function

LV diameter and wall thickness were measured from the parasternal long axis view. LV mass was estimated using the Devereux formula (Lang et al., 2015) and indexed to body surface area. LV diastolic function was assessed from early peak (E) and atrial (A) transmitral flow velocities. Tissue Doppler Imaging (TDI) velocities were assessed at the mitral annular level from apical views. Peak E’ (i.e., average of the septum and lateral wall) was used as less load‐dependent indexes of LV relaxation (Nagueh et al., 2016). The LV ejection fraction was assessed using the Simpson's biplane method.

#### 2.5.2 Left ventricular strains

LV global longitudinal strain (GLS), an index of myocardial systolic function (Lang et al., 2015), was obtained as previously detailed (Grandperrin, Schuster, et al., 2022).

#### 2.5.3 Left atrial morphology and function

LA morphology was assessed by measuring the LA volume index (LAVI) using the biplane area‐length method (Thomas et al., 2019). Deformations analysis of the LA was performed from the apical four chambers view according to the methodological recommendations of ref. (Vieira et al., 2014). The LA endocardial border was manually traced using a point‐and‐click technique, starting from the septal and tracing to the lateral aspect of the mitral valve annulus. The minimal width of the region of interest was selected. LA longitudinal strain curves were used to assess reservoir function during LV systole, LA conduct function during early passive LV filling, and LA booster pump function during active contraction of the LA (Vieira et al., 2014).

### 2.6 Statistical analysis

Sample size calculation was therefore based on the experience of our laboratory and on the work of (Arbab‐Zadeh et al., 2014) in endurance training. Based on LVM modification, sample size calculation estimated that a total number of 10 participants in each group would be required, considering and effect size of 1.2 with a statistical power of 0.8 and an alpha of 0.05. Considering a potential withdrawal of participants due to the stringent protocol (3 times a week over 16 weeks) we finally enrolled 17 participants in each group. All values were expressed as mean ± standard deviation (SD). Statistical analyses were performed using Statview 5.0 software (SAS Institute Inc., USA). Normality of the distribution was checked using the Kurtosis and Skewness coefficient and the homogeneity of the variances was assessed by the Bartlett test. A two‐way analysis of variance (ANOVA) was used to compare groups and time effects and the Holm–Sidak post‐Hoc test was used to compare differences between each evaluation. Percentage of change was calculated by comparing differences between pre and post‐test in women and men according to the recommendations in sport sciences (Buchheit, 2016; Winter et al., 2014). According to these recommendations and to the methodological paper published by Cousineau et al. (2021) (Cousineau et al., 2021), the magnitude of within‐group differences (pre to post‐test) was expressed as effect size (ES) using Cohen's paired *d*. They were calculated by dividing the mean difference (between pre and post‐test) by the average of their standard deviations for each group. Confidence interval (CI) for Cohen's paired *d* was calculated for each parameter. They were then classified as small (0–0.59), moderate (0.6–1.19), large (1.2–1.99), and very large (>2.0) (Hopkins et al., 2009).

## 3 RESULTS

Twenty‐four participants (12 women and 12 men) completed more than 90% of the 48 training sessions (95.0 ± 3.7% and 94.7 ± 3.3% of sessions completed for women and men, respectively) and were included in the analysis. As shown in Figure 1, withdrawals were mainly due to lack of time to perform three training sessions a week (8 participants) or due to long‐time business trip (2 participants). Baseline characteristics of the study population are presented in Table 1. Body mass was lower in women than men at baseline and remained constant during the ST program. Systolic and diastolic blood pressures were also lower in women than men at baseline and remained unchanged in both groups throughout the ST program (systolic blood pressure: from 118 ± 8–119 ± 5 mmHg in women, ES: 0.2 CI [−0.4–0.5] vs. 127 ± 7–125 ± 5 mmHg in men, ES: 0.3 CI [−0.2–0.6]; Diastolic blood pressure: from 77 ± 6–77 ± 3 mmHg in women, ES: 0.1 CI [−0.1–0.3] vs. 83 ± 7–80 ± 5 mmHg in men, and ES: 0.3 CI [−0.2–0.5]). Heart rate was higher in women at baseline and remained unchanged (from 65 ± 7–66 ± 8 bpm in women, ES: 0.1 CI [−0.1–0.3] vs. 58 ± 6–61 ± 8 bpm in men, and ES: 0.3 CI [−0.2–0.5]).

**TABLE 1:** Clinical characteristics and arterial blood pressures at baseline.

	Men	Women	*p*‐value
	Mean ± SD	Mean ± SD	
Clinical characteristics			
Age, years	27 ± 4	29 ± 6	0.4
Height, cm	176 ± 5	167 ± 6	<0.0001
Body mass, kg	84.6 ± 13.1	62.1 ± 4.8	<0.0001
Body mass index, kg.m^−2^	24.1 ± 3.6	23.0 ± 2.6	0.3
Arterial blood pressures			
Systolic blood pressure, mmHg	127 ± 7	118 ± 8	0.01
Diastolic blood pressure, mmHg	83 ± 7	77 ± 6	0.01

### 3.1 Strength performance

Maximal performances at the bench press and squat are presented in Table 2 and Figure 2. The 1‐RM on bench press was significantly higher at baseline in men compared to women and increased significantly every 4 weeks in both groups. Of note, the increase was higher in women (from 26 ± 6 kg to 43 ± 6 in women, ES:2.7 CI [1.7–4.1] vs. 68 ± 19 kg to 88 ± 17 kg in men, ES:1.1 CI [0.6–1.6]; Interaction = 0.03). The 1‐RM for the squat was higher in men than women at baseline, but increased similarly for both groups throughout the training program (Figure 2B, ES: 2.8 CI [1.7–4.3] and 1.9 CI [1.1–2.9] in women and men, respectively).

**TABLE 2:** Strength performance, body composition and cardiac morphology and function at baseline and after the ST program in women and men.

	Baseline	After the ST program
	Mean ± SD	Mean ± SD
Strength performances		
1‐RM bench press, kg		
Men	67.9 ± 19.1	87.9 ± 17.5
Women	26.5 ± 6.2^##^	42.9 ± 5.8^##^
1‐RM squat, kg		
Men	82.5 ± 20.9	118.7 ± 16.4
Women	47.1 ± 14.7^##^	83.3 ± 10.5^##^
Body composition		
Lean mass, kg		
Men	36.9 ± 3.9	37.6 ± 3.9
Women	24.4 ± 2.6^##^	24.9 ± 2.3^##^
Fat mass, %		
Men	23.0 ± 6.1	22.6 ± 5.7
Women	28.2 ± 4.1^##^	26.0 ± 4.4^##^
Cardiac morphology and function		
LVMi, g.m^−2^		
Men	89.1 ± 9.6	109.2 ± 12.9***
Women	67.1 ± 12.2^###^	82.5 ± 12.4^###^***
E wave, cm.s^−1^		
Men	71.7 ± 16.8	77.6 ± 15.5
Women	84.1 ± 8.1^#^	89.1 ± 11.8^#^
A wave, cm.s^−1^		
Men	35.2 ± 6.9	29.5 ± 6.1*
Women	42.8 ± 10.5^##^	40.2 ± 7.9^##^*
E′ mean, cm.s^−1^		
Men	10.49 ± 1.81	10.60 ± 1.37
Women	11.25 ±1.54	11.28 ± 1.12
EF, %		
Men	62.2 ± 4.4	63.9 ± 2.9*
Women	63.4 ± 3.5	65.3 ± 2.4*
GLS, %		
Men	−19.56 ± 1.76	−18.65 ± 1.39
Women	−20.69 ± 1.44^#^	−20.35 ± 1.26^#^
LAVI, mL.m^−2^		
Men	23.9 ± 3.9	25.3 ± 4.2
Women	20.3 ± 1.7^##^	20.9 ± 1.8^##^
LA reservoir function, %		
Men	30.5 ± 7	30.8 ± 9.1
Women	38.1 ± 6.7^#^	36.4 ± 8.9^#^

Abbreviations: EF, ejection fraction; GLS, global longitudinal strain; LA, left atrium; LAVI, left atrium volume index; LV, left ventricular; LVMi, left ventricular mass index.

# Significantly different from men (#, *p* < 0.05; ##, *p* < 0.01; ###, *p* < 0.001)/* significantly different from pretest (*, *p* < 0.05; ***, *p* < 0.001).

**FIGURE 2:**
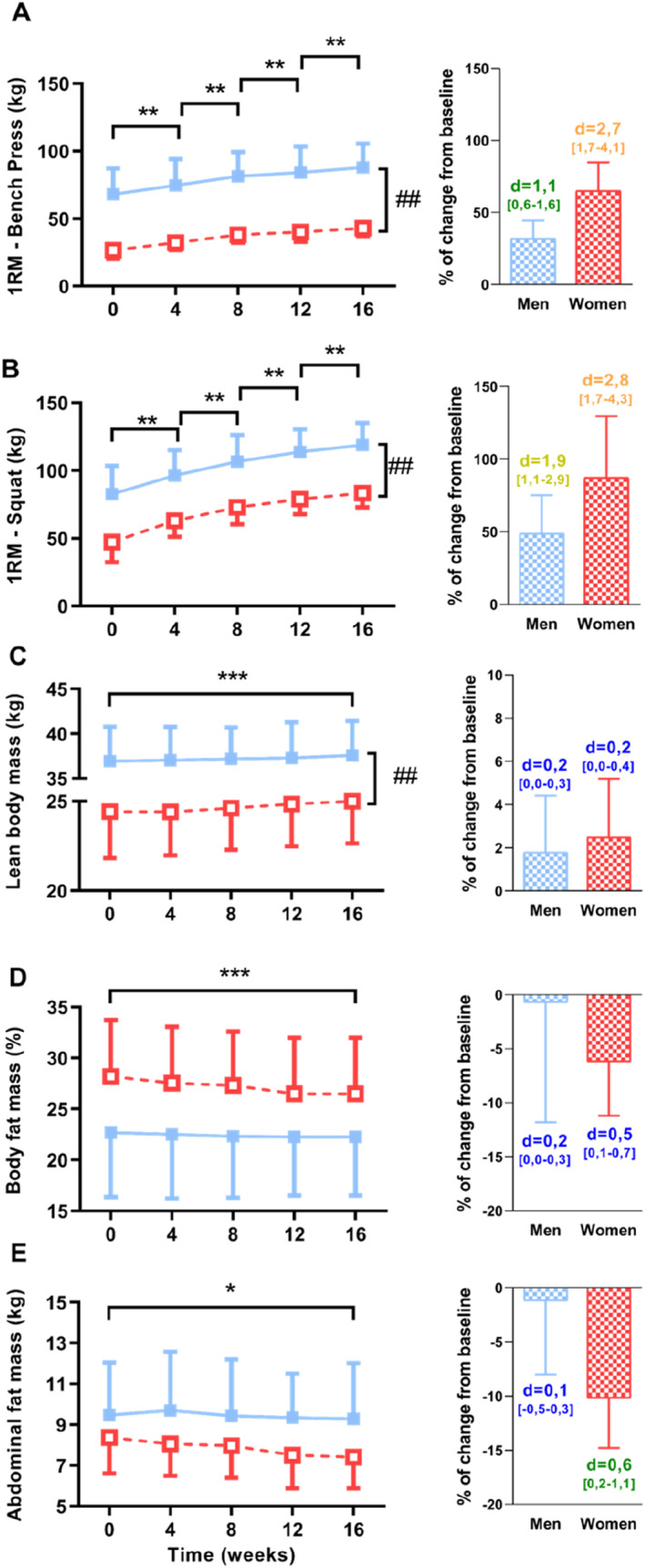
Alterations of strength performance and body composition during the 16‐week of strength‐training program including (A) 1‐RM for the bench press, (B) 1‐RM for the squat, (C) lean body mass, (D) percentage of body fat and (E) abdominal fat mass. On the right‐side, histograms represent the percentage of change from baseline in both groups with effect size (number above histogram represents Cohen's d with [confidence intervals], blue: small effect, green: moderate effect, yellow: large effect and orange: very large effect). *, significantly different from other evaluation (*, *p* < 0.05; **, *p* < 0.01; ***, *p* < 0.001); ##, significant differences between groups at baseline (*p* < 0.01).

### 3.2 Body composition

Parameters of body composition are presented in Table 2 and Figure 2. Lean body mass was lower in women than men at baseline and increased to a similar extent in both groups during the ST program (Figure 2C, ES: 0.2 CI [0.0–0.4] and 0,2 CI [0.0–0.3] in women and men, respectively). The percentage of body fat mass was higher in women than men at baseline and decreased in both groups (Figure 2D, ES: 0.5 CI [0.1–0.7] and 0.2 CI [0.0–0.3] in women and men, respectively). Abdominal fat mass also decreased in both groups (Figure 2E, ES: 0.6 CI [0.2–1.1] and 0.1 CI [−0.5–0.3] in women and men, respectively).

### 3.3 Left ventricular morphology

**FIGURE 3:**
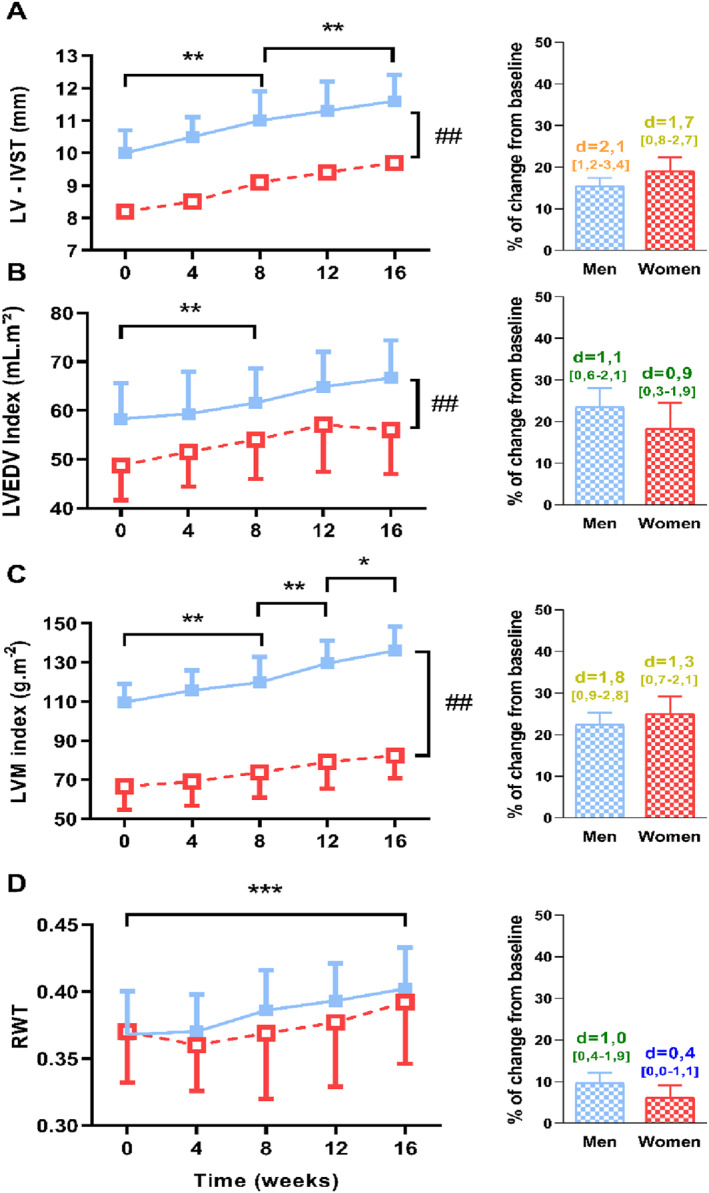
Alteration of left ventricular morphological parameters over the 16‐week strength‐training program. (A) Left ventricular interventricular septum thickness (IVST), (B) Left ventricular end‐diastolic volume indexed to body surface area, (C) Left ventricular mass indexed to body surface area (LVMi) and (D) relative wall thickness (RWT). On the right‐side, histograms represent the percentage of change from baseline in both groups with effect size (number above histogram represents Cohen's d with [confidence intervals], blue: small effect, green: moderate effect, yellow: large effect and orange: very large effect). *, significantly different from other evaluations (*, *p* < 0.05; **, *p* < 0.01; ***, *p* < 0.001); ##, group differences (*p* < 0.01).

LV morphological parameters are presented in Table 2 and Figure 3. At baseline, the interventricular septum thickness (Figure 3A), LV end‐diastolic volume index (Figure 3B) and the LV mass index (Figure 3C) were significantly lower in women than men. Interestingly, LV end‐diastolic volume index and LV mass index progressively increased throughout the training program in a similar extent in both groups (LV end‐diastolic diameter: ES: 0.9 CI [0.3–1.9] in women and 1.1 CI [0.6–2.1] in men; LV mass index: ES: 1.3 CI [0.7–2.1] in women and 1.8 CI [0.9–2.8] in men, Figure 3B,C). Similarly, interventricular septum thickness progressively increased in both groups (ES: 1.7 CI [0.8–2.7] in women and 2.1 CI [1.2–3.4] in men). Relative wall thickness (Figure 3D), which were similar between women and men at baseline, progressively increased in both groups, but in a greater extent in men than women (ES: 0.4 CI [0.0–1.1] in women and 1.0 CI [0.4–1.9] in men). Finally, the ratio between LV diastolic volume and LA volume was unchanged throughout the protocol in both groups (from 2.43 ± 0.47–2.48 ± 0.43 in women, ES: 0.5 CI [0.1–0.8] and from 2.47 ± 0.39–2.58 ± 0.42 in men, ES: 0.7 CI [0.3–1.0]).

### 3.4 Left ventricular function

**FIGURE 4:**
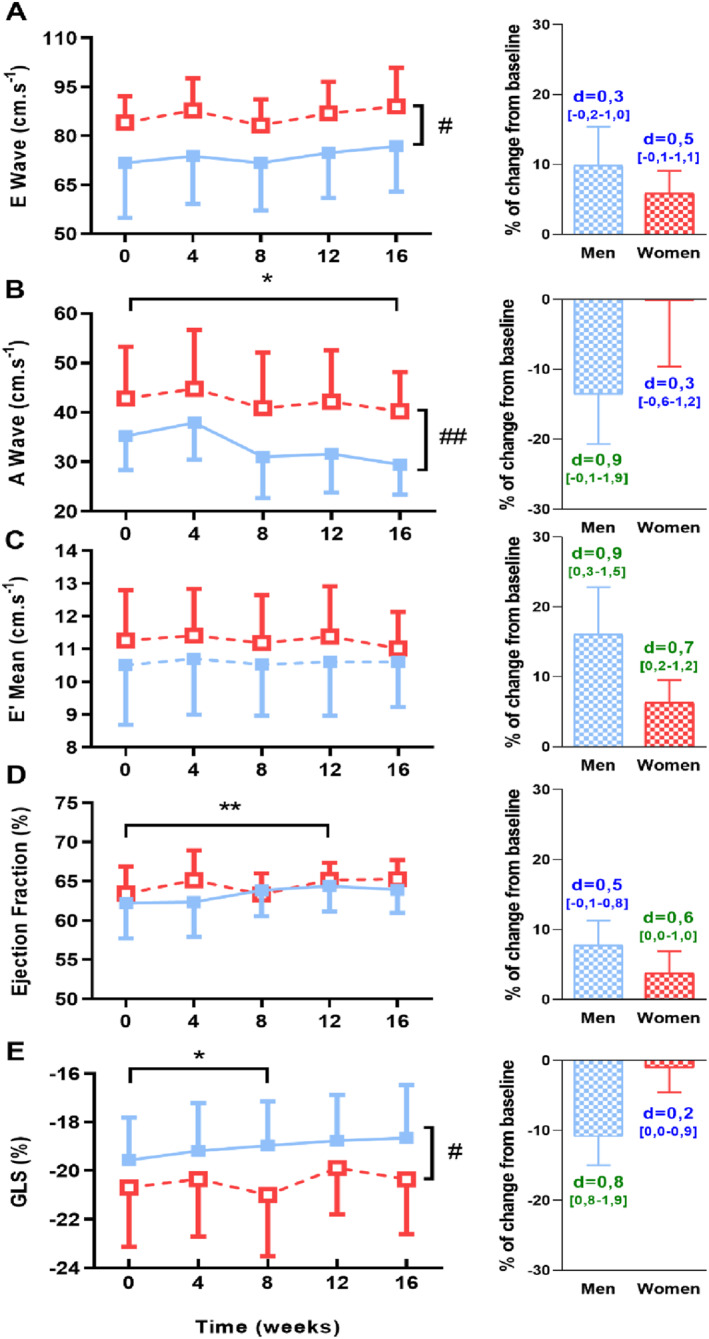
Alterations of left ventricular function over the 16‐week strength‐training program. (A) transmitral Doppler E wave, (B) transmitral Doppler A wave, (C) Tissue Doppler E′ mean on the septum and lateral wall, (D) ejection fraction (EF) and (E) global longitudinal strain (GLS). On the right‐side, histograms represent the percentage of change from baseline in both groups with effect size (number above histogram represents Cohen's d with [confidence intervals], blue: small effect, green: moderate effect). *, significantly different from other evaluations (*, *p* < 0.05; **, *p* < 0.01); #, significant group differences (#, *p* < 0.05; ##, *p* < 0.01).

The parameters of LV function are presented in Table 2 and Figure 4. At baseline, the E wave (Figure 4A), A wave (Figure 4B) and GLS (Figure 4C) were higher in women than men, whereas E′ mean and EF were similar between both groups. The E wave (figure 4A) remained unchanged throughout the 16‐week ST program in both groups (ES: 0.5 CI [−0.1–1.1] in women and 0.3 CI [−0.2–1.0] in men). The A wave (Figure 4B) decreased progressively in both women and men (ES: 0.3 CI [−0.6–1.2] in women and 0.9 CI [−0.1–1.9] in men). The E'_mean_ (Figure 4C) remained statistically unchanged during the protocol (ES: 0.7 CI [0.2–1.2] in women and 0.9 CI [0.3–1.5] in men). In regard to systolic function, EF (Figure 4D) increased significantly from week 0 to week 12 in a similar range between women and men (ES: 0.6 CI [0.0–1.0] in women and 0.5 CI [−0.1–0.8] in men). GLS (Figure 4E) remain unchanged throughout the protocol in both groups (ES: 0.2 CI [0.0–0.9] in women and 0.8 CI [0.8–1.9] in men).

### 3.5 Left atrial morphology and function

**FIGURE 5:**
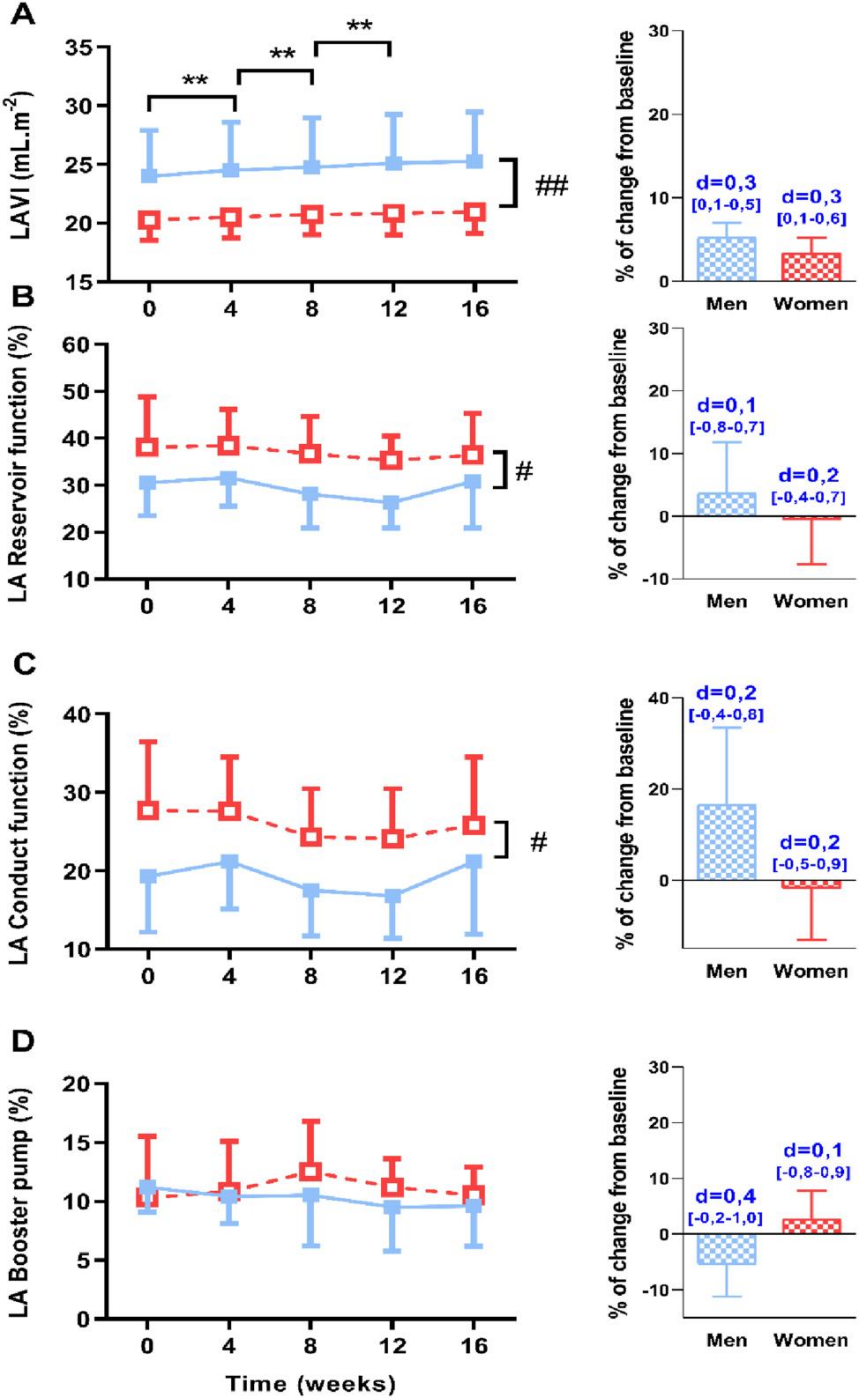
Alteration of left atrial morphology and function over the 16‐week strength‐training program. (A) Left atrium volume index (LAVI), (B) reservoir function, (C) conduct function and (D) booster pump. On the right‐side, histograms represent the percentage of change from baseline in both groups with effect size (number above histogram represents Cohen's d with [confidence intervals], blue: small effect). **, significantly different from other evaluations (**, *p* < 0.01); #, significant group differences (*p* < 0.05).

LA morphology and function are presented in Table 2 and Figure 5. At baseline, LAVI (Figure 5A) was significantly lower in women than in men, while reservoir (Figure 5B) and conduct (Figure 5C) functions were significantly higher in women than men. The LAVI progressively increased to a similar extent in both groups from week 0 to week 12 (ES: 0.3 CI [0.1–0.6] in women and ES: 0,3 CI [0.1–0.5] in men). Reservoir (Figure 5B), conduct (Figure 5C), and booster pump (Figure 5D) functions remained unchanged in both groups throughout the training program.

## 4 DISCUSSION

To the best of our knowledge, this is the first study to evaluate the effect of sex on the impact of a supervised ST program on performance, body composition, and cardiac remodeling in previously untrained subjects. The main findings of this study were that (1) women exhibited similar or even higher strength performance increases than men, (2) both women and men showed similar alterations in body composition, with decrease of body and abdominal fat mass and increase of lean body mass, and (3) both women and men had cardiac remodeling characterized by an increase in LV mass associated with an alteration of LV function.

### 4.1 Impact of the ST program on the muscular strength in women and men

Few longitudinal training studies were conducted in women, making our understanding of their adaptations induced by ST limited, especially in previously untrained subjects. The first important finding of this study was that when a standardized ST program in line with the recommendation of the ACSM was proposed in women and men, women had similar and significant improvements of muscular strength compared to men. Early attempts to assess the efficiency of ST in women concluded that, relative to men, moderate strength gains were acquired (Mayhew et al., 1974; Wells et al., 1963; Wilmore, 1974). However, more recent studies that used training protocols with similar volume, intensity, and duration to those typically employed by men indicated that strength gains were similar in women compared to men (Häkkinen, Kallinen et al., 1998, Häkkinen, Pakarinen et al., 1998; Lemmer et al., 2000). Interestingly, our results strongly support these observations since the increase of muscular strength was similar or even significantly greater on bench press in women compared to men. This unexpected result might be explained by significantly lower strength values at baseline in women compared to men. Interestingly, although sex differences exist compared with men in terms of lean body mass, muscle cross‐sectional area, percentage of fast muscle fibers (Deschenes & Kraemer, 2002; Fournier et al., 2022), and of absolute testosterone production in response to ST (Weiss et al., 1983), they did not lead to lower adaptations in the early phase of ST. We hypothesized that the 10‐fold lower concentrations of testosterone in women could have led to a lower stimulation of the IGF‐1/PI3K/Akt hypertrophy signaling pathway, and consequently to lower muscular adaptations. It has been well‐demonstrated that strength improvements were most likely mediated by a combination of physiological adaptations in the neuromuscular system, including improvements in neural activation, greater cross‐sectional area, and changes in muscle architecture and morphology (Kraemer et al., 2002). The similar adaptation between women and men found in our study can probably be explained by the predominance of neural adaptation during the early stage of training rather than myofibril hypertrophy. Interestingly, a recent study provides information about the impact of menstrual cycle combined with oral contraceptive utilization on power and strength (Wikström‐Frisén et al., 2017), a parameter that could partly explain our results even if further investigations in the field are needed. In our study, all women used oral contraceptive and were evaluated every 4 weeks and then probably at the same period of their cycle. Finally, review based of more than one hundred studies reported that muscular strength increased approximatively by 40% in untrained, 20% in moderately trained, 16% in trained, and 5% in elite participants over various training periods (Kraemer et al., 2002). Our results are in accordance with more than 40% progression on 1‐RM bench press and squat in our groups.

### 4.2 Impact of the ST program on the body composition in women compared to men

Considering the link between body fat mass—and more especially abdominal fat mass—and cardiovascular health (Westcott, 2012), understanding the impact of a ST program on body composition is of major interest. In the present study, we used impedancemetry, a non‐invasive procedure which has been validated against gold standard method (Ling et al., 2011) and which can provide additional values on abdominal fat mass. The increase of lean body mass represents a direct effect of ST since a recent meta‐analysis revealed similar results (Morton et al., 2018). Indeed, based on 49 studies and more than 1900 participants, authors showed that ST led to an increase in fiber cross sectional area and as a consequence of muscle mass. At the onset of this muscle fiber hypertrophy, not only the enhancement of protein synthesis is mediated by post‐transcriptional regulation (Kadi et al., 2004) but also via the incorporation of additional myonuclei in muscle fiber by satellite cells. Indeed, satellite cells represents muscle precursor cells with the ability to provide additional myonuclei to another fiber in response to ST (Kadi et al., 2004). Moreover, we found a decrease not only in fat mass but also in abdominal fat mass consecutive to ST. Even if alteration seemed quite low, probably due of the short‐term duration of the protocol, there were in accordance with a previous review reporting a reduction of body fat mass from 1% to 9% following ST programs of various durations (American College of Sports Medicine, 2009) in healthy populations. This is particularly true when the program includes high‐volume and intensity training with brief between‐set rest intervals (Deschenes & Kraemer, 2002) as proposed in our study. Interestingly, in our study, ES on body and abdominal fat mass indicated that the impact of our ST program was higher in women compared to men. Research studies designed to measure the effects of ST on fat mass were usually carried out in overweight and/or obese participants and have often been confounded by concomitant dietary changes and/or weight loss. Nevertheless, Hurley et al. (2011) (Hurley et al., 2011) identified increased resting metabolic rate, improved insulin sensitivity, and enhanced sympathetic activity as possible mechanisms by which ST could decrease intra‐abdominal fat stores in women. Since excessive body and abdominal fat mass is associated with risk factors such as elevated plasma cholesterol, plasma glucose and resting blood pressure (Westcott, 2012), ST programs in respect to the recommendation of the ACSM appears to be an effective strategy to modulate body composition in order to promote health benefits in both women and men.

### 4.3 Impact of the ST program on the LV morphological remodeling in women and men

The present study provided additional data on the “physiological” adaptation of cardiac chambers in women, since very few data are available in this specific field. In ST athletes, numerous investigations reported morphological and functional heart adaptations after several years of training (Haykowsky et al., 2002). However, the impact of ST on cardiac function in previously untrained subjects has been inadequately studied, and it remains unclear whether these cardiac adaptations are similar in both women and men. Interestingly, we observed a LV enlargement in women and men, characterized by an increase in LV wall thickness, volume, and mass. This result highlighted that women can exhibit similar myocardial adaptations to ST training compared to men. Once again, we hypothesized that low level of testosterone could have led to lower cardiac remodeling consecutive to ST. Of note, Marsh et al. (1998) (Marsh et al., 1998) observed similar level of androgen receptors in the heart of men and women. Moreover, our results supported that these morphological adaptations occurred very early during the training program (i.e., after 4 weeks). This is in agreement with previous longitudinal studies using cardiac magnetic resonance imaging and underlying that cardiac enlargement occurred after only several weeks of endurance (Matsuo et al., 2014) or strength (Spence et al., 2011; Vogelsang et al., 2008) training. Our data strongly supported that these early cardiac adaptations were also effective in women from the beginning of the ST program.

It has been proposed that LV remodeling in ST athletes is a consequence of an important increase in arterial pressure and cardiac afterload during effort (Lentini et al., 1993; MacDougall et al., 1992). An increase in cardiac afterload stimulates hypertrophic signaling pathways via sarcomere expansion (McMullen et al., 2007). Mac Dougall et al. (MacDougall et al., 1992) reported a significant correlation between intensity of the training and arterial blood pressure during effort. In our study, ST program was composed of exercises performed at 70% of the individual 1‐RM, which can be considered as high. Moreover, as explained previously these high‐intensity exercises lead to important increase in testosterone levels which also could explain this morphological adaptation (Weiss et al., 1983). When such high‐intensity ST program was performed three times a week and includes exercises involving large muscles, the training load appears sufficient to induce cardiac morphological adaptations after several weeks, in both women and men.

Finally, we also observed an increase in the relative wall thickness from 0.37 to 0.40 in men and from 0.37 to 0.39 in women, suggesting a progressive concentric remodeling throughout the ST program. Nevertheless, values obtained at the end of the ST program remained lower than those observed in athletes who practiced ST over a long period of time, with values from 0.43 to 0.46 (see for review Haykowsky et al., 2002).

### 4.4 Impact of the ST program on the LV functional remodeling in women and men

To assess potential subtle alterations in LV function, we used not only standard but also tissue Doppler and regional 2D‐strain echocardiography. 2D‐strain analyses give the possibility to get inside into the regional LV mechanics in order to assess myocardial regional function. As previously described, at baseline, women had higher E wave, A wave and GLS, probably due to their higher heart rate (Shim et al., 2011; Sun et al., 2013). It has been proposed that the increase of heart rate led to a phosphorylation of protein kinase A and C which enhance inotropy and lusitropy by influencing the calcium handling process (Ramirez‐Correa et al., 2010). Interestingly, diastolic function was progressively altered during the ST program in both women and men, with an increase in the E/A ratio resulting from a decrease in peak A only. The E′ mean, a less‐load independent index of LV relaxation, was unchanged throughout the ST program in both women and men, traducing an absence of improvement of LV relaxation, as previously described in athletes (Beaumont et al., 2017). Indeed, the relaxation of the myocardium seemed unchanged at rest after strength or endurance training, but appeared increased during exercise (Beaumont et al., 2017). Similarly, EF was constant during the ST program in women and men, a result also observed previously (Haykowsky et al., 2002). The GLS, which is now well‐recognized as a more sensitive tool to assess LV systolic function, was unchanged throughout the protocol. To our knowledge, we were the first to assess the GLS adaptation consecutive to ST in previously untrained subjects. The normal EF and GLS despite LV hypertrophy was a clinically relevant point since in pathological hypertrophic conditions these parameters were altered as well as in hypertrophic cardiomyopathy (Grandperrin, Schuster, et al., 2022). Taken together, our data highlighted that LV function remained unchanged throughout a 16‐week ST program in both women and men.

### 4.5 Impact of the ST program on the LA morphology and function in women and men

Atrial function is an integral part of cardiac function that has been often neglected. The LA modulates LV filling and cardiac performance through its roles as a reservoir during LV systole and as a conduct and booster pump during LV diastole. In the present study, LA morphology was evaluated using LAVI and function was assessed using 2D‐strain longitudinal analysis. At baseline, women had smaller LAVI compared to men, an observation consistent with previous studies that might be explained by higher amounts of body fat mass or smaller chest dimensions (Wernstedt et al., 2002). Interestingly, the LA progressively enlarged from the beginning of the ST program in a similar extent in women and men, as previously reported (Giraldeau et al., 2015). The ratio between LV end‐diastolic volume and LA volume remain unchanged during the ST program, highlighting a harmonious development of these two cavities. To the best of our knowledge, this is the first study highlighting the LA morphological adaptation to ST in previously untrained women and men. Finally, LA reservoir, conduit and booster pump functions remained unchanged throughout the ST program in both groups. LA parameters might be clinically relevant for risk stratifications, being related to adverse cardiac events, such as atrial fibrillation, heart failure, and mortality (Sengupta et al., 2014). The morphological remodeling of the LA without modification of function was in line with our previous results reported in high‐level strength‐trained athletes and confirmed that ST is not associated with atrial dysfunction (Grandperrin, Schnell, et al., 2022). To our knowledge, no information was previously reported on LA function consecutive to longitudinal ST.

### 4.6 Strengths and limitations of the present study

The strength of our study was to propose a similar supervised 4 months ST program in terms of relative intensity, duration, and type of exercise in previously sedentary women and men. The 24 subjects included in the statistical analysis completed at least 90% of the training sessions. Despite the relatively small size of our groups, the sample was still superior to the sample needed to achieve a sufficient statistical power for our main outcomes. Several limitations were inherent to our study. Firstly, because we did not include a control group, we cannot exclude the possibility that some of the changes observed in this study were a function of time and day‐to‐day variability. However multiple previous investigations showed that measures of cardiac morphology and function and exercise performance are highly reproductible and constant over longitudinal periods (Hastings et al., 2012; Perhonen et al., 2001; Shibata et al., 2010). Secondly, the echocardiographic analysis focused on the LV and LA without evaluation of the right chambers. Finally, body composition and echocardiographic parameters could be biased in women due to biological variations induced by the menstrual cycle. Nevertheless, George et al. (2000) (George et al., 2000) reporting no meaningful differences in functional parameters between follicular and luteal phases in healthy women.

## 5 CONCLUSION

Our 16‐week ST program conducted in line with the recommendations of the ACSM induced an increase in strength performance, a beneficial effect on body composition, and a morphological cardiac remodeling in previously sedentary women and men. There was an enlargement of both the LV and LA, very early during the ST program, with few impact on LV function. Our data bring new evidence that when a rigorously similar and standardized ST program, in accordance with the recommendations of the ACSM, was prescribed in women and men, women responded to a similar manner compared to men in terms of strength performance, body composition, and cardiac adaptation. These findings are of major interest to promote ST in women with an objective of health and performance.

## CONFLICT OF INTEREST STATEMENT

There is no conflict of Interest to declare.
